# Development and optimization of a novel nanocarrier SabiWhite-loaded ethosomal gel for targeted skin inflammation complicated by multidrug-resistant pathogens

**DOI:** 10.3389/fcimb.2025.1640799

**Published:** 2025-07-22

**Authors:** Gaofeng Shi, Yun Guo, Minlie Yang

**Affiliations:** Burn and Trauma Treatment Center, Affiliated Hospital of Jiangnan University, Wuxi, Jiangsu, China

**Keywords:** SabiWhite, ethosomes, anti-inflammatory, multidrug-resistant pathogens, nanocarrier, topical therapy, combinational treatment

## Abstract

**Background:**

This study aimed to develop and evaluate SabiWhite-loaded ethosomes (SW-ETH) for topical application, focusing on improving stability, biocompatibility, and therapeutic efficacy. Ethosomal formulations are known for their enhanced drug delivery properties, making them suitable for skin inflammation.

**Methods:**

The SW-ETH formulations were developed utilizing an adapted cold preparation technique. A 3² factorial design was used to optimize phospholipid concentration and ethanol content, and their impact on vesicle size and entrapment efficiency (EE%) was assessed. Structural characterization of SabiWhite was performed using melting point determination, Fourier-Transform Infrared Spectroscopy (FTIR), and X-ray Diffraction (XRD). *In vitro* drug release was assessed using a Franz diffusion cell, and anti-inflammatory and skin irritation studies were performed on Wistar rats.

**Results:**

SabiWhite exhibited a melting point of 96°C and characteristic FTIR peaks, confirming its identity and purity. XRD analysis revealed its crystalline nature, while ethosomal formulations showed a shift to an amorphous state. The optimized SW-ETH formulation (SW-ETH 6) had a vesicle size of 184.4 nm, an EE% of 92.5%, and a zeta potential of -13.50 mV, indicating stable and uniform vesicles. *In-vitro* drug release from SW-ETH 6 showed a sustained release profile with 93.12% drug release over 24 hours. *In vivo*, SW-ETH demonstrated significant anti-inflammatory effects with 36.17% edema inhibition at 150 minutes, comparable to Diclofenac gel (41.92%). No skin irritation was observed, and the formulation was classified as non-irritant. Stability tests confirmed minimal changes in appearance, viscosity, and drug content over 120 days at different storage conditions.

**Conclusion:**

SW-ETH demonstrated effective drug encapsulation, enhanced anti-inflammatory activity, and excellent biocompatibility, making it a promising candidate for topical therapy. Further clinical validation is warranted to confirm its therapeutic potential.

## Introduction

Skin inflammation is a prevalent dermatological condition characterized by redness, swelling, itching, and pain, which can significantly impair the quality of life (Tampa et al., 2022). It encompasses a wide spectrum of disorders, including dermatitis, psoriasis, and eczema, often triggered by infections-, often involving multidrug-resistant (MDR) pathogens like *Staphylococcus aureus*, *Pseudomonas aeruginosa*, *and Acinetobacter baumannii*, which exacerbate inflammation, delay healing, and compromise therapeutic outcomes also allergens, autoimmune reactions, and environmental factors such as ultraviolet (UV) radiation, pollutants, and chemical irritants (Kantor and Silverberg, 2017). In particular, methicillin-resistant *Staphylococcus aureus* (MRSA) colonization has been reported in up to 60% of patients with chronic eczematous dermatitis, where it perpetuates inflammation by releasing superantigens and proteases (Kim et al., 2019). The immune cells, including keratinocytes, macrophages, and mast cells, primarily drive the inflammatory response by activating and releasing pro-inflammatory cytokines such as tumor necrosis factor-alpha (TNF-α), interleukin-6 (IL-6), and interleukin-1beta (IL-1β) (Arango Duque and Descoteaux, 2014). Chronic inflammation disrupts skin barrier function, promoting oxidative stress, collagen degradation, and delayed wound healing, which underscores the need for effective therapeutic interventions (Mamun et al., 2024). The conventional treatments for skin inflammation include corticosteroids, nonsteroidal anti-inflammatory drugs (NSAIDs), and immunomodulators such as calcineurin inhibitors. While corticosteroids are highly effective in reducing inflammation, their long-term use is associated with adverse effects, including skin atrophy, telangiectasia, and increased susceptibility to infections (Calabrese et al., 2022). NSAIDs, though widely used, exhibit limited efficacy in chronic conditions and may cause irritation and hypersensitivity reactions. Furthermore, biologics targeting specific inflammatory pathways, such as monoclonal antibodies against TNF-α (e.g., adalimumab) and IL-17 (e.g., secukinumab), have shown promise in severe inflammatory disorders but are associated with high costs and systemic immunosuppressive effects (Schwartz et al., 2017). The limitations of existing therapies highlight the urgent need for safer, cost-effective, and targeted alternatives to manage skin inflammation effectively. Curcumin, the bioactive component of Curcuma longa, has been extensively studied for its anti-inflammatory, antioxidant, and wound-healing properties (Sharifi-Rad et al., 2020). However, its poor aqueous solubility, rapid degradation, and low bioavailability limit its therapeutic application. SabiWhite, a standardized curcumin derivative, has emerged as a promising alternative due to its enhanced stability, increased skin permeability, and superior pharmacological profile. Studies have demonstrated that SabiWhite exerts potent anti-inflammatory effects by inhibiting nuclear factor-kappa B (NF-κB) activation, reducing oxidative stress, and downregulating pro-inflammatory cytokines (Sureshbabu et al., 2025). Additionally, its depigmenting properties make it a suitable candidate for treating inflammatory hyperpigmentation disorders. Despite its advantages, the topical delivery of SabiWhite remains a challenge due to its hydrophobic nature and limited skin retention. Nanocarrier-based drug delivery systems have revolutionized dermatological therapy by improving drug solubility, penetration, and sustained release (Kang et al., 2024). Ethosomes, lipid-based nanovesicles composed of phospholipids, ethanol, and water, offer a unique advantage for transdermal drug delivery (Verma and Pathak, 2010). The presence of ethanol enhances lipid fluidity, allowing ethosomes to penetrate deeper skin layers while maintaining high drug encapsulation efficiency (Hmingthansanga et al., 2022). Ethosomal formulations have been successfully utilized for delivering bioactive compounds in the treatment of psoriasis, eczema, and wound healing (Abdulbaqi et al., 2016). Given the promising attributes of ethosomes, incorporating SabiWhite into an ethosomal system could overcome its solubility and permeability limitations, thereby enhancing its anti-inflammatory efficacy. The application of nanotechnology in dermatology is a rapidly expanding field, with increasing research efforts focused on optimizing nano-based drug delivery systems for inflammatory skin disorders (DeLouise, 2012). Globally, recent studies have demonstrated the effectiveness of ethosomal carriers for delivering herbal and synthetic drugs, offering improved therapeutic outcomes. In China, research on traditional herbal medicines and their nanoformulations has gained significant momentum, driven by the growing interest in integrating modern pharmaceutical technology with Traditional Chinese Medicine (TCM) (Zhang et al., 2024). Several studies have explored curcumin-based formulations for treating inflammatory skin conditions, yet the incorporation of SabiWhite into an ethosomal system remains largely unexplored. Given the high prevalence of dermatitis and psoriasis in China, developing an effective, stable, and targeted topical therapy could offer substantial clinical and commercial benefits (Wang et al., 2024). This research focuses on the development and optimization of a SabiWhite-loaded ethosomal gel for treating skin inflammation. Using a factorial design, the formulation will be optimized for entrapment efficiency, stability, and skin permeability. Key evaluations include vesicle size, polydispersity index, zeta potential, and morphology. Franz diffusion studies will assess drug release and kinetics, while carrageenan-induced paw edema in Wistar rats will evaluate anti-inflammatory efficacy. Additionally, biocompatibility, safety, and stability will be examined. By integrating nanoformulation with herbal pharmacotherapy, this study aims to offer a safer and more effective alternative to conventional treatments.

## Methods

The study was conducted in accordance with the guidelines of the Institutional Animal Ethics Committee (IAEC), and all experimental procedures involving animals were approved (Approval No.: Z201710; ethical committee of Wuxi Third People’s Hospital). The study adhered to the ethical principles outlined by the Committee for Control and Supervision of Experiments on Animals (CPCSEA) and followed the ARRIVE guidelines for reporting animal research. Adequate measures were taken to minimize animal suffering and ensure humane handling.

### Chemicals and reagents

All chemicals used in this study were of laboratory-grade. SabiWhite, a standardized curcumin derivative, was procured from Merck, USA. Ethanol and Phospholipon 90G were also sourced from Merck, while the remaining reagents were obtained from certified suppliers in China.

### Identification and characterization of SabiWhite

SabiWhite was characterized using multiple analytical techniques to confirm its identity, purity, and physicochemical properties. The capillary method was used to determine the melting point of SabiWhite, a standard approach for assessing purity (Loron et al., 2021). A finely powdered sample of SabiWhite was placed in a sealed glass capillary tube and subjected to controlled heating using a digital melting point apparatus. The temperature at which the substance transitioned from a solid to a liquid state was recorded. A sharp and narrow melting range indicates high purity, whereas a broader or lower melting range suggests the presence of impurities. Fourier-Transform Infrared Spectroscopy (FTIR) was conducted to identify the functional groups and chemical bonds present in SabiWhite (Soury et al., 2023). The sample was prepared by mixing a small quantity of SabiWhite with potassium bromide (KBr) and compressing the mixture into a transparent pellet. The pellet was then analyzed using an FTIR spectrometer, where infrared radiation was passed through the sample. The resulting spectra were examined to detect characteristic absorption peaks corresponding to functional groups such as hydroxyl (-OH), carbonyl (C=O), and aromatic rings. FTIR analysis is essential for verifying the molecular structure of SabiWhite and assessing its compatibility in formulation systems. The crystalline nature and solid-state properties of SabiWhite were evaluated using XRD. A finely ground sample was placed in the sample holder of an X-ray diffractometer, and monochromatic X-rays were directed at the sample under controlled conditions (Soury et al., 2023). The diffracted X-rays produced a pattern of peaks, which were analyzed to determine the crystal lattice structure and degree of crystallinity. Sharp, well-defined peaks in the XRD pattern indicate a highly crystalline material, whereas broad and diffuse peaks suggest an amorphous nature. XRD analysis provided crucial insights into the structural integrity and phase purity of SabiWhite.

### Preparation and characterization of SabiWhite-loaded nanoethosomes

SabiWhite-loaded ethosomes were prepared using a modified cold method originally described by Touitou et al (Touitou et al., 2000). Briefly, Phospholipon^®^ 90G and SabiWhite (5 mg/mL) were dissolved in ethanol and stirred at 700 rpm using a magnetic stirrer (RCT Basic, IKAMAG, IKA, Germany) at 30 ± 1°C in an airtight container. Phosphate-buffered saline (PBS, pH 6.4) was added dropwise (200 µL/min) under continuous stirring at the same temperature. The resulting suspension was stirred for an additional 10 minutes and stored at 4°C overnight for vesicle swelling. To achieve uniform vesicle size, the suspension was probe-sonicated for 5 minutes at 4°C using an ultrasonic sonicator, followed by sequential filtration through sterile 0.45 µm and 0.20 µm syringe filters (10 cycles). The final dispersion was stored at 4°C for further characterization. A 3² factorial design was used for formulation optimization, with phospholipid concentration (X1) and ethanol content (X2) as independent variables, while vesicle size (Y1) and entrapment efficiency (Y2) were selected as dependent variables (**Supplementary Table 1**). The amount of SabiWhite remained constant across all formulations. Entrapment efficiency (EE%) was determined via ultracentrifugation. A 2 mL aliquot of the ethosomal suspension was centrifuged at 20,000 rpm for 3 hours at 4°C (Kubota Model 7000, Japan). The supernatant was collected, diluted, and analyzed using a UV-visible spectrophotometer (Lab India UV-3200, India) to quantify unencapsulated SabiWhite. EE% was calculated as: $\%EE=\left(\frac{T-S}{T}\right)X100$ , where T is the total amount of SabiWhite in the formulation, and S is the unencapsulated amount in the supernatant. The optimized formulation was selected based on maximizing entrapment efficiency and achieving a vesicle size within the 100–200 nm range.

### Characterization of SabiWhite-loaded nanoethosomes

#### Vesicle size, size distribution, and zeta potential

The vesicle size, polydispersity index (PDI), and zeta potential of SW-ETH formulations were measured using a Zetasizer Nano ZS (Malvern Instruments Ltd., Malvern, UK). Samples were appropriately diluted with Milli-Q water to avoid multiple scattering effects and analyzed at 25 ± 0.5°C. Dynamic light scattering (DLS) at the laser wavelength (633 nm) and scattering angle (173°) was used to determine vesicle size and size distribution, while electrophoretic light scattering measured the zeta potential to assess formulation stability. The results were expressed as mean ± standard deviation (SD) from three independent measurements followed by the entrapment efficiency (EE%) of SabiWhite within ethosomal vesicles was determined as mentioned above.

#### Vesicle shape and morphology

The morphology and structural integrity of SW-ETH vesicles were analyzed using optical microscopy and transmission electron microscopy (TEM). Optical microscopy was performed using a Radical Scientific Equipments Pvt. Ltd. RXL-4T microscope at 20× magnification to observe the preliminary vesicular structure before sonication. The images were captured at a magnification of 100,000x, and we added that the accelerating voltage used was 80 kV. A drop of diluted ethosomal suspension was placed on a clean glass slide, covered with a coverslip, and examined for vesicle uniformity (Corona et al., 2023). For detailed structural analysis, TEM was used to assess vesicle shape and lamellarity. A drop of the optimized formulation was placed on a carbon-coated copper grid and allowed to air dry, forming a thin film. A 1% phosphotungstic acid (PTA) solution was added as a negative stain, and the excess stain was carefully removed with filter paper. The sample was examined under a transmission electron microscope, and images were captured to evaluate vesicle morphology, size consistency, and membrane structure. Quantitative assessment of vesicle size from TEM images was performed using ImageJ software, with at least 100 vesicles measured per sample.

### *In-vitro* drug release study of SabiWhite-loaded nanoethosomes

The *in vitro* drug release of SabiWhite from SW-ETH formulations was evaluated using a Franz diffusion cell (diffusion area: 3.14 cm²) with a receptor chamber volume of 20 mL. A dialysis membrane (MWCO 12–14 kDa) was pre-soaked in phosphate-buffered saline (PBS, pH 5.5) overnight at 25 ± 0.5°C before being positioned between the donor and receptor compartments (Salamanca et al., 2018). To simulate non-occluded skin conditions, the donor compartment was left open. The receptor medium was maintained at 37 ± 0.5°C and continuously stirred at 100 rpm using a magnetic stirrer (Remi Equipment Pvt Ltd., India) to ensure uniform mixing. At predetermined time intervals (0.5, 1, 2, 3, 4, 6, 8, and 24 hours), 1 mL aliquots were withdrawn from the receptor compartment and immediately replaced with an equal volume of fresh PBS (pH 5.5) to maintain sink conditions. The withdrawn samples were analyzed for SabiWhite content using a UV-visible spectrophotometer at 284 nm. All experiments were performed in triplicate, and the results were expressed as mean ± standard deviation (SD).

### Drug release kinetics

To elucidate the drug release mechanism, the obtained release data were fitted to Zero-Order kinetic models (Alshahrani et al., 2024): (constant release rate independent of drug concentration):C=C_0_-K_0_t. Where C is the cumulative drug release, C_0_ is the initial drug concentration, K_0_ is the zero-order rate constant, and t is time. A plot of cumulative drug release versus time was analyzed for linearity. All kinetic models were analyzed by calculating the correlation coefficient (R^2^), and the best-fitting model was determined based on the highest R² value.

### *In-vivo* anti-inflammatory and skin irritation studies of SabiWhite-loaded nanoethosomes

The anti-inflammatory efficacy of SW-ETH was evaluated using a carrageenan-induced paw edema model in Wistar albino rats (Ben Khedir et al., 2016). The study was conducted in compliance with OECD guidelines and received approval from the institutional animal ethics committee. Twelve female, nulliparous, and non-pregnant Wistar rats (200–250 g, aged 8–12 weeks) were randomly divided into four groups (n=3 per group). The control group (Group I) received carrageenan injection without any treatment, while the reference group (Group II) was pre-treated with Diclofenac gel (2 mg/paw equivalent to 10 mg/cm²) 30 minutes before carrageenan injection. Group III was treated with SW-ETH gel (2 mg/paw), and Group IV received a placebo gel. Carrageenan (0.1 mL, 1% w/v) was injected into the plantar region of the right hind paw to induce inflammation, and formulations were applied topically 30 minutes prior to the injection. The paw volume was measured using a digital caliper at specific time points (30, 60-, 90-, 120-, and 150 minutes post-injection).

The percentage inhibition of edema was calculated using the equation:

**Percent edema inhibition** = $\left[1-\left(\frac{VT}{V0}\right)X100\right]$ where V_T_ represents the edema volume in the treated group and V_0_ represents the edema volume in the control group.

### Skin irritation study

A skin irritation study was conducted following OECD Guideline 404. Three female Wistar albino rats were used, and their dorsal fur was removed 24 hours before testing. A 0.5 g dose of SW-ETH gel was applied to a 6 cm² area using a gauze patch secured with a semi-occlusive dressing. The patch was removed after four hours, and the test site was observed for erythema and edema at 60 minutes, 24 hours, 48 hours, and 72 hours post-application. Dermal reactions were graded based on OECD-defined criteria, ranging from 0 (no irritation) to 4 (severe irritation) (**Supplementary Table 2**). The primary irritation index (PII) was calculated as the mean sum of erythema and edema scores. Based on PII values, skin responses were classified as non-irritant (0.0), negligible irritant (0.1–0.4), slight irritant (0.41–1.9), moderate irritant (2.0–4.9), or severe irritant (5.0–8.0).

### Stability determination

The stability of the optimized SW-ETH gel was evaluated over 90 days under three different storage conditions: refrigeration (4 ± 2°C), room temperature (25 ± 2°C), and accelerated conditions (37 ± 2°C). The formulation was stored in amber glass containers to minimize light-induced degradation. Samples were analyzed at predetermined intervals for changes in physical appearance, viscosity, pH, drug content, and spreadability (Kirtane et al., 2022). Viscosity measurements were conducted using a Brookfield viscometer (spindle #1, 50 rpm), while pH was determined using a calibrated digital pH meter. Drug content was analyzed via UV-visible spectrophotometry at 284 nm. Spread ability was evaluated using the circular plate method, wherein a 2 cm diameter gel sample was placed between two glass plates, and the final spread diameter was measured. The formulation was considered stable if no significant changes in these parameters were observed over 90 days.

### Statistical analysis

All experiments were performed in triplicate (n=3). Given the exploratory nature of this formulation development study and in accordance with the journal’s statistical guidelines for small sample sizes, non-parametric permutation tests were employed for group comparisons instead of traditional ANOVA. This approach makes no assumptions about data distribution and is specifically recommended for studies with n<5 (DDDT Editorial, June 2023) (Panos and Boeckler, 2023). For all analyses, *p*-values were generated through 10,000 random permutations of the dataset using R software (version 4.2.1). Effect sizes with 95% confidence intervals are reported where applicable. All data are presented as mean ± SD with permutation-based p-values <0.05 (p(perm)=0.021) considered significant.

## Results

### Characterization analysis

**Figure 1:**
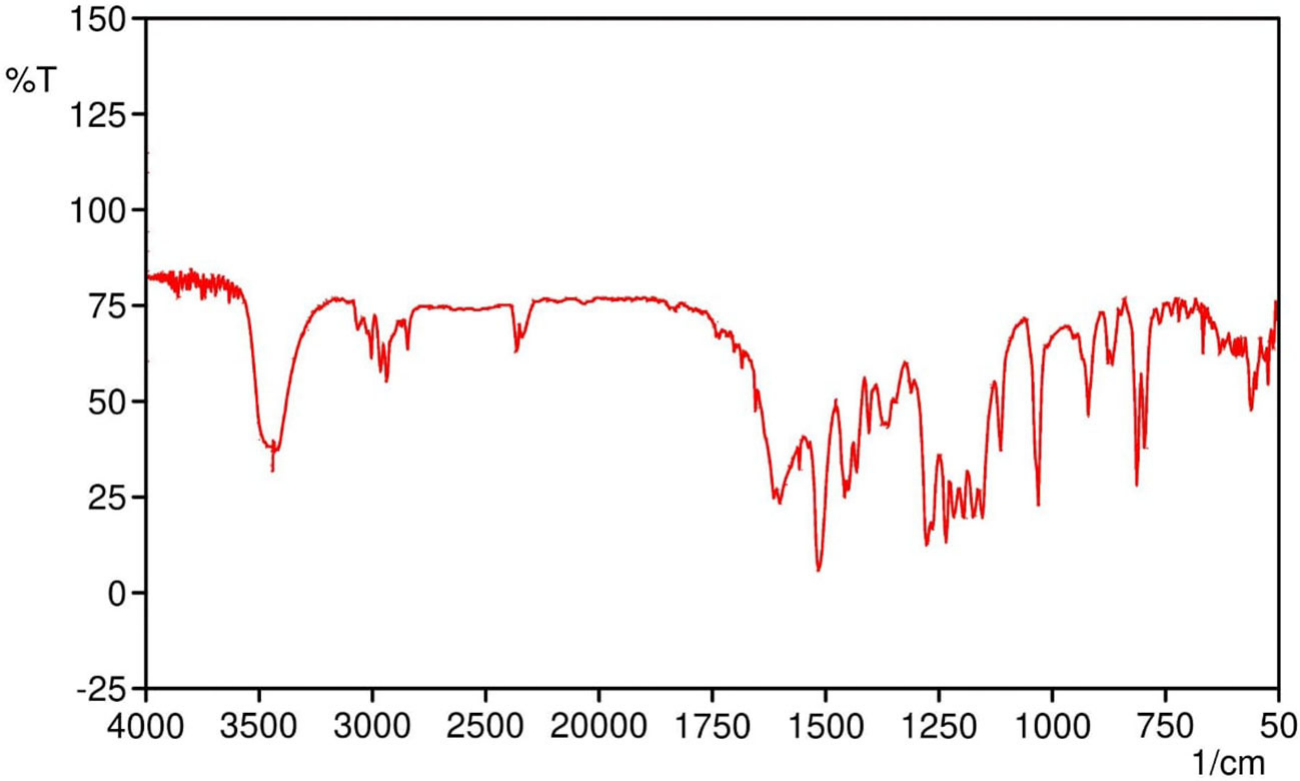
FTIR spectrum of SabiWhite showing characteristic functional groups. The prominent peaks at 3421 cm^-1^ (O-H stretching), 3200–3000 cm^-1^ (C-H stretching), 2933 cm^-1^ (OCH_3_ group stretching), 1601 cm^-1^ (C=O stretching), 1457 cm^-1^ (C-H bending of methyl groups), 1277 cm^-1^ (C-O stretching), and 1032 cm^-1^ (C-O-CH_3_ stretching) are identified, confirming the molecular structure and functional groups present in SabiWhite.

The melting point of SabiWhite was determined to be 96°C, aligning closely with previously reported values, thereby confirming the identity and purity of the compound. The structural identity of SabiWhite was further validated through Fourier-transform infrared (FTIR) spectroscopy. The FTIR spectrum (**Figure 1**) exhibited characteristic absorption peaks at 3421 cm^-1^ (O-H stretching), 3200–3000 cm^-1^ (C-H stretching), 2933 cm^-1^ (OCH_3_ stretching), 1601 cm^-1^ (C=O stretching), 1457 cm^-1^ (C-H bending of methyl groups), 1277 cm^-1^ (C-O stretching), and 1032 cm^-1^ (C-O-CH_3_ stretching). These peaks correspond to functional groups intrinsic to SabiWhite, confirming its molecular structure and chemical integrity.

**Figure 2:**
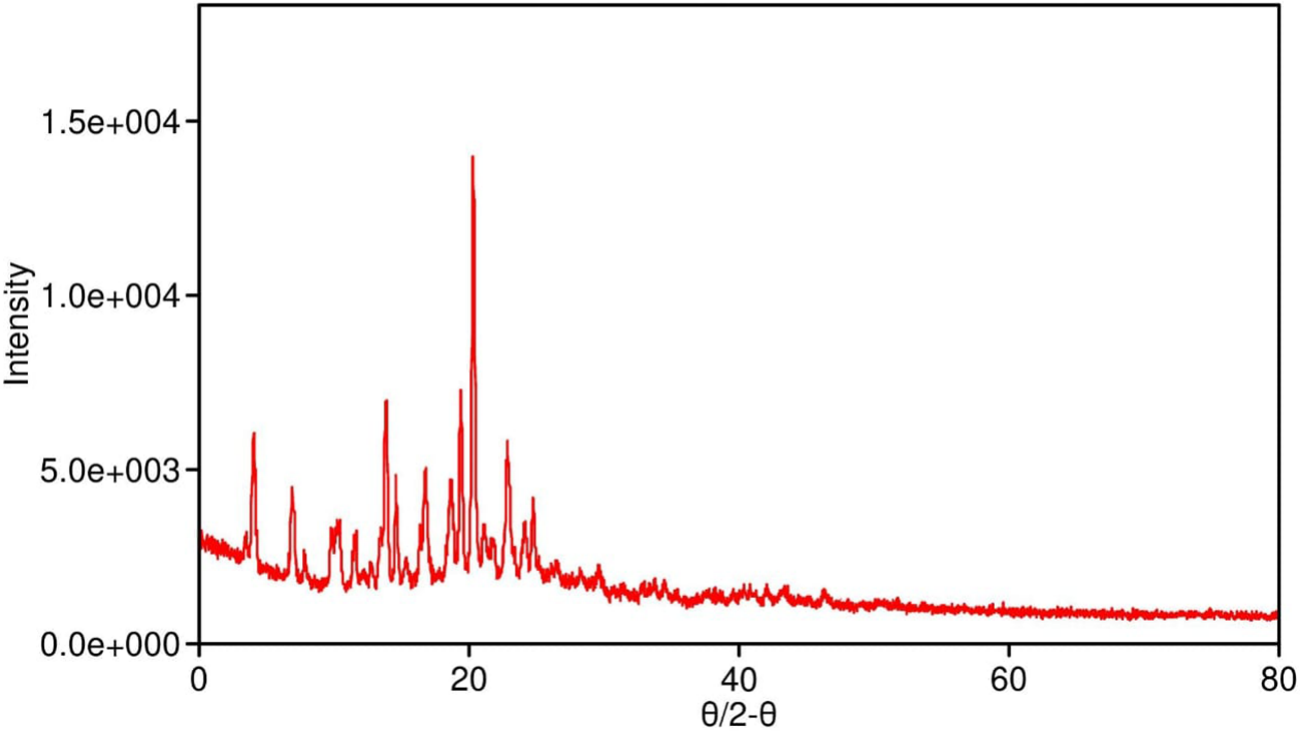
XRD pattern of pure SabiWhite, phospholipid, and SabiWhite-phospholipid mixture. Pure SabiWhite exhibits a sharp diffraction peak at 18.560°, indicating its crystalline nature. The phospholipid shows a broad peak at 21.649°, typical of an amorphous structure. In the physical mixture, the diffraction peak of SabiWhite is retained, suggesting that its crystalline structure remains unchanged. However, in ethosomal formulations, the distinct diffraction peak of SabiWhite is integrated into the lipid matrix, transitioning into an amorphous or disordered state.

X-ray diffraction (XRD) analysis of pure SabiWhite (**Figure 2**) revealed a distinct and sharp diffraction peak at 18.560°, indicative of its crystalline nature. In contrast, the phospholipid exhibited a broad diffraction peak at 21.649°, characteristic of an amorphous structure. The physical mixture of SabiWhite and phospholipid retained the diffraction peak of pure SabiWhite, suggesting that its crystalline structure remained unaltered. However, in the ethosomal formulations, the diffraction peak of SabiWhite was no longer distinctly observed, indicating its integration into the lipid matrix and a transition to an amorphous or disordered state. This structural transition from crystalline to amorphous could potentially enhance the bioavailability of SabiWhite. Amorphous forms of compounds generally exhibit higher solubility compared to their crystalline counterparts, which may improve their dissolution rate and skin permeability. However, this transition may also influence the chemical stability of the compound, as amorphous forms can sometimes be more prone to degradation. Further stability studies would be required to fully assess the impact of this transition on the long-term stability of SabiWhite in its ethosomal formulation.

### Characterization of SW-ETH formulations

The formulation of SW-ETH was successfully achieved through the modified cold method. The optimization of vesicle size and entrapment efficiency (EE%) was carried out using a 3² factorial design, with phospholipid concentration (X1) and ethanol content (X2) as independent variables. The resulting formulations exhibited vesicle sizes ranging from 100.6 nm to 271.6 nm and entrapment efficiencies from 77.1% to 94.8%. **Table 1** summarizes the vesicle size, entrapment efficiency (EE%), polydispersity index (PDI), and zeta potential (ZP) of all formulations. The formulation SW-ETH 6, with 3% phospholipid and 45% ethanol, achieved the best results in terms of both entrapment efficiency (92.5%) and vesicle size (184.4 nm). This formulation was selected for further optimization and characterization.

**Table 1:** Vesicle size and entrapment efficiency of SW-ETH formulations.

Formulation code	Phospholipid (% w/v) (X_1_)	Ethanol (% v/v) (X_2_)	Vesicle size (nm) (Y_1_)	Entrapment efficiency (% EE) (Y_2_)	PDI	Zeta potential (mV)
SW-ETH 1	2	25	120.2	77.1	0.1	-8.8
SW-ETH 2	2	35	113.5	81.5	0.15	-9.7
SW-ETH 3	2	45	100.6	85.3	0.16	-12.5
SW-ETH 4	3	25	213.1	82.6	0.18	-8.9
SW-ETH 5	3	35	203.7	89.2	0.2	-9.75
SW-ETH 6	3	45	184.4	92.5	0.25	-13.5
SW-ETH 7	4	25	271.6	86.3	0.2	-8.95
SW-ETH 8	4	35	247.1	91.2	0.2	-10.1
SW-ETH 9	4	45	220.4	94.8	0.27	-14.1

### ANOVA and regression analysis

Analysis of variance (ANOVA) for both vesicle size and entrapment efficiency was conducted to evaluate the statistical significance of the models. The model F-values were found to be highly significant for both responses (vesicle size and entrapment efficiency), with p-values < 0.0001 for vesicle size and 0.0014 for entrapment efficiency. The results of ANOVA for both responses are shown in **Table 2**. Furthermore, regression analysis was conducted for both vesicle size and entrapment efficiency. The quadratic model was found to be the most suitable for both responses, with R² values of 0.9993 for vesicle size and 0.9945 for entrapment efficiency, indicating a strong fit between the observed and predicted data (**Table 3**).

**Table 2:** ANOVA results for SW-ETH formulation responses.

Source	Response Y_1_: vesicle size	Response Y_2_: % entrapment efficiency
Model F-Value	30153.29	262.76
*p*-value (Prob > F)	< 0.0001	0.0014
X_1_ (Phospholipid)	27310.51	134.62
X_2_ (Ethanol)	1650.04	118.28
X_1_X_2_ (Interaction)	249.64	0.02
X_1_² (Quadratic)	924.5	8.38
X_2_² (Quadratic)	18.6	1.47

**Table 3:** Regression analysis results for SW-ETH.

Model	Standard deviation	R²	Adjusted R²	Predicted R²	PRESS	CV (%)	Remark
Vesicle Size (Y_1_)	Linear	14.22	0.9598	0.9464	2787.14	7.64	Suggested
	2FI	13.88	0.9681	0.9489	3868.18	7.46	
	Quadratic	2.58	0.9993	0.9982	225.53	1.38	
Entrapment Efficiency (Y_2_)	Linear	1.37	0.9572	0.9429	1.37	1.58	Suggested
	2FI	1.5	0.9573	0.9316	1.5	1.73	
	Quadratic	0.69	0.9945	0.9854	0.69	0.8	

### Impact of variables on vesicle size and entrapment efficiency

**Figure 3:**
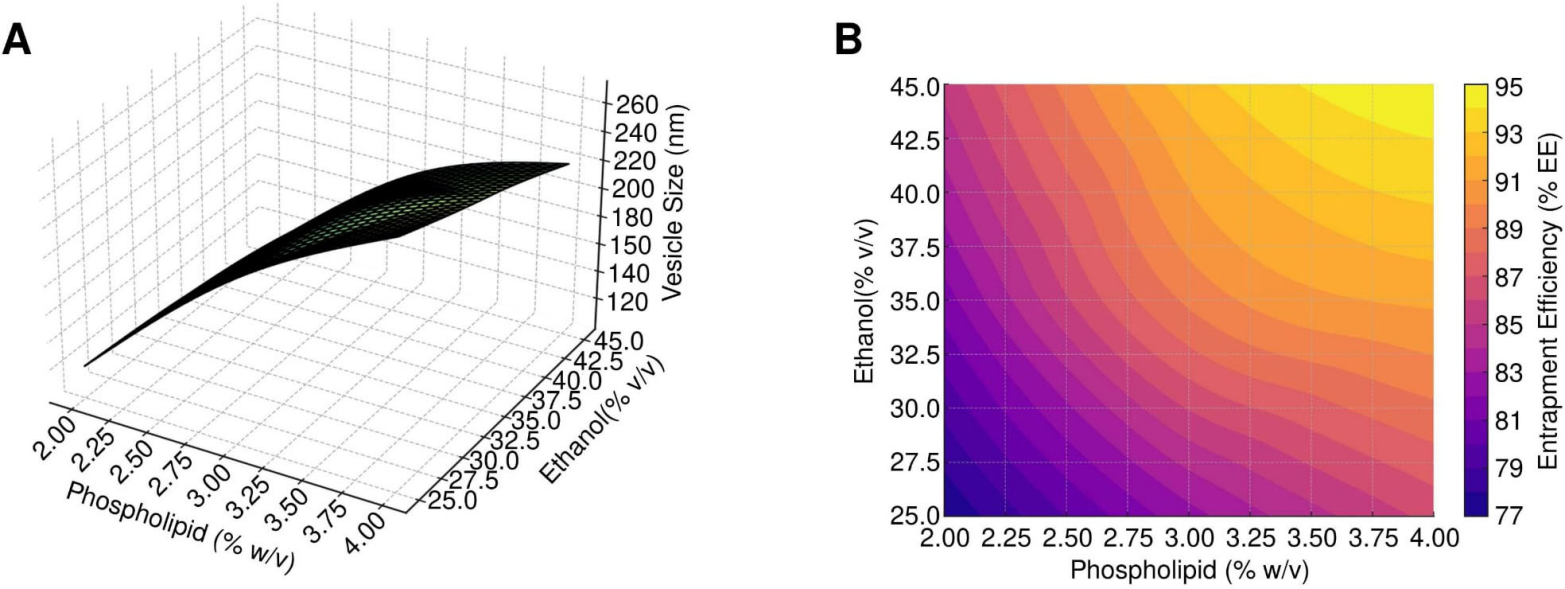
Response surface and contour analysis of SW-ETH formulations. **(A)** 3D-Response Surface, 2D-Contour, and Perturbation Plots illustrating the impact of phospholipid and ethanol on vesicle size. **(B)** 3D-Response Surface, 2D-Contour, and Perturbation Plots depicting the effect of phospholipid and ethanol on entrapment efficiency.

The effect of phospholipid and ethanol concentration on vesicle size and entrapment efficiency was analyzed using response surface plots, 2D contour plots, and perturbation graphs. For vesicle size, an increase in phospholipid concentration from 2% to 4% resulted in larger vesicles, while a higher ethanol concentration reduced vesicle size, as depicted in the 3D response surface and 2D contour plots (**Figures 3A, B**). Similarly, for entrapment efficiency, both higher phospholipid and ethanol concentrations positively influenced EE%, as illustrated in **Figure 3B**.

### Optimization of SW-ETH formulation

Numerical optimization was employed to identify the optimal formulation with desired attributes. The optimized formulation, SW-ETH 6, exhibited a vesicle size of 184.4 nm and an entrapment efficiency of 92.5%. These results align closely with the predicted values, confirming the accuracy of the model. The optimized formulation was further characterized for PDI and zeta potential, which were found to be 0.25 and -13.50 mV, respectively, indicating a stable and uniform formulation.

### Vesicle size, PDI, and zeta potential

**Figure 4:**
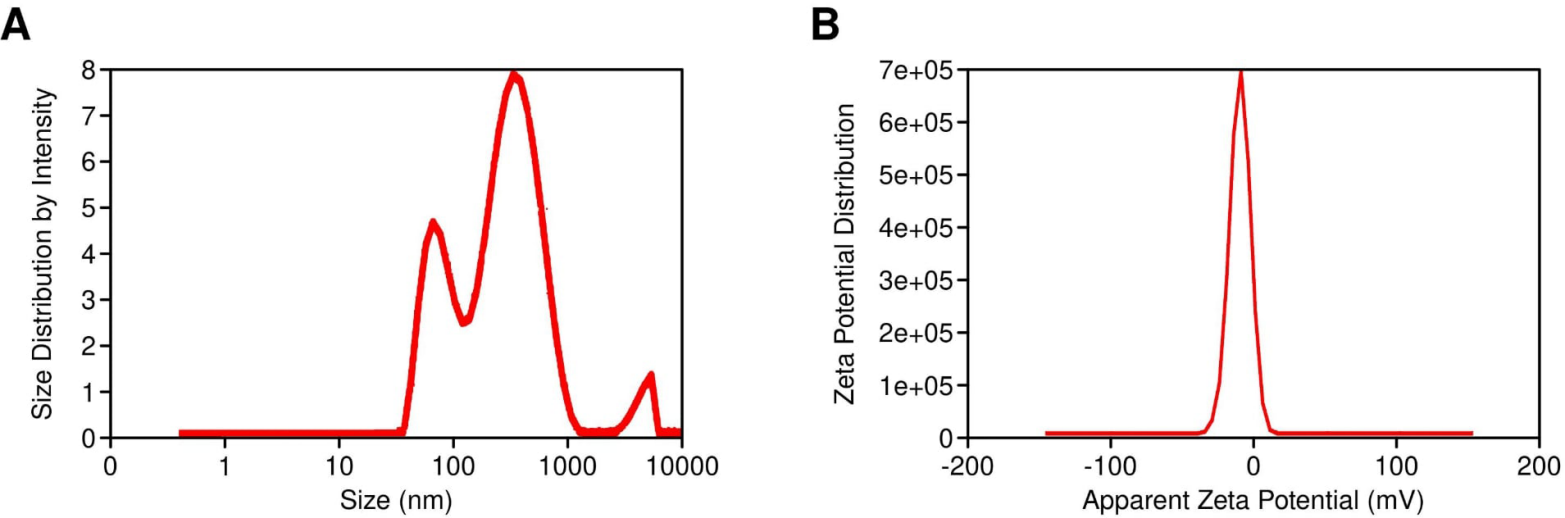
Characterization of OPT-SW-ETH formulations. **(A)** Vesicle size distribution and polydispersity index (PDI) of OPT-SW-ETH. **B**; Zeta potential distribution of OPT-SW-ETH.

The vesicle size of the ethosomal formulation ranged from 120 nm to 220 nm, with the optimized SabiWhite-loaded ethosomal formulation (OPT-SW-ETH) exhibiting a size of 184.40 nm (**Figure 4A**). The polydispersity index (PDI), a key indicator of size uniformity, ranged from 0.10 to 0.27, with OPT-SW-ETH showing a PDI of 0.25, confirming a homogeneous vesicle distribution (**Figure 4A**). A PDI below 0.3 is generally considered acceptable (Danaei et al., 2018), supporting the uniform nature of the formulation. Zeta potential (ZP) analysis was performed to assess the stability of the ethosomal vesicles. The ZP values ranged from -8.80 mV to -14.10 mV, with OPT-SW-ETH displaying a ZP of -13.50 mV (**Figure 4B**). The negative surface charge can be attributed to the presence of ethanol and phospholipid, which contribute to vesicle stability by inducing repulsive forces that prevent aggregation. Higher absolute values of ZP indicate better colloidal stability, reinforcing the integrity of the optimized formulation.

### Vesicle shape and morphology

**Figure 5:**
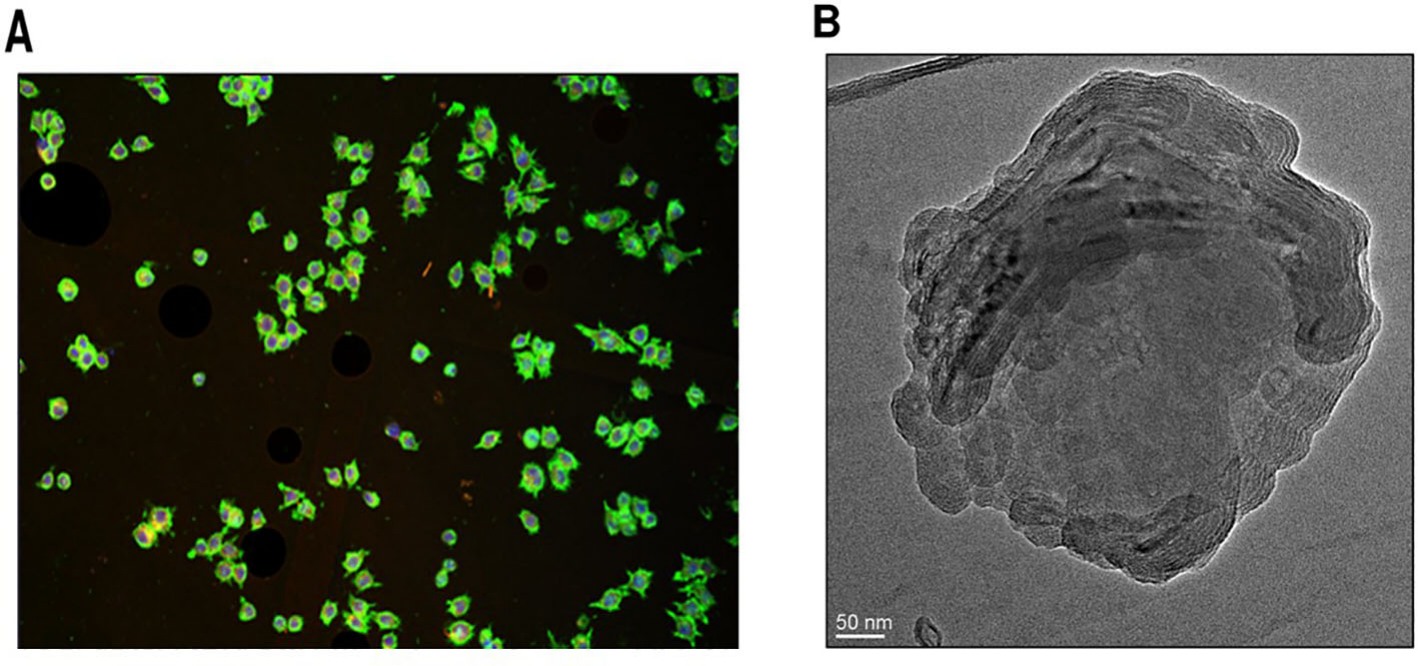
Morphological analysis of SW-ETH vesicles. **(A)** Optical microscopy image showing spherical shape and multi-lamellarity. **(B)** TEM image confirming the presence of unilamellar vesicles with nanometer-scale size.

Optical microscopy revealed vesicles with a spherical shape and multi-lamellarity, with no signs of aggregation or fusion (**Figure 5A**). A drop of diluted SW-ETH suspension was placed on a glass slide, covered with a coverslip, and examined under an RXL-4T microscope (20× magnification). The images confirmed the presence of discrete, well-formed vesicles. A drop of the optimized formulation was deposited on a carbon-coated copper grid and left to air-dry, followed by staining with 1% phosphotungstic acid (PTA). The TEM images confirmed the presence of unilamellar vesicles in the nanometer range with slightly irregular shapes (**Figure 5B**). Quantitative vesicle size analysis from TEM images was conducted using ImageJ software, measuring at least 100 vesicles per sample to ensure statistical accuracy.

### *In-vitro* drug release profile of SW-ETH

**Figure 6:**
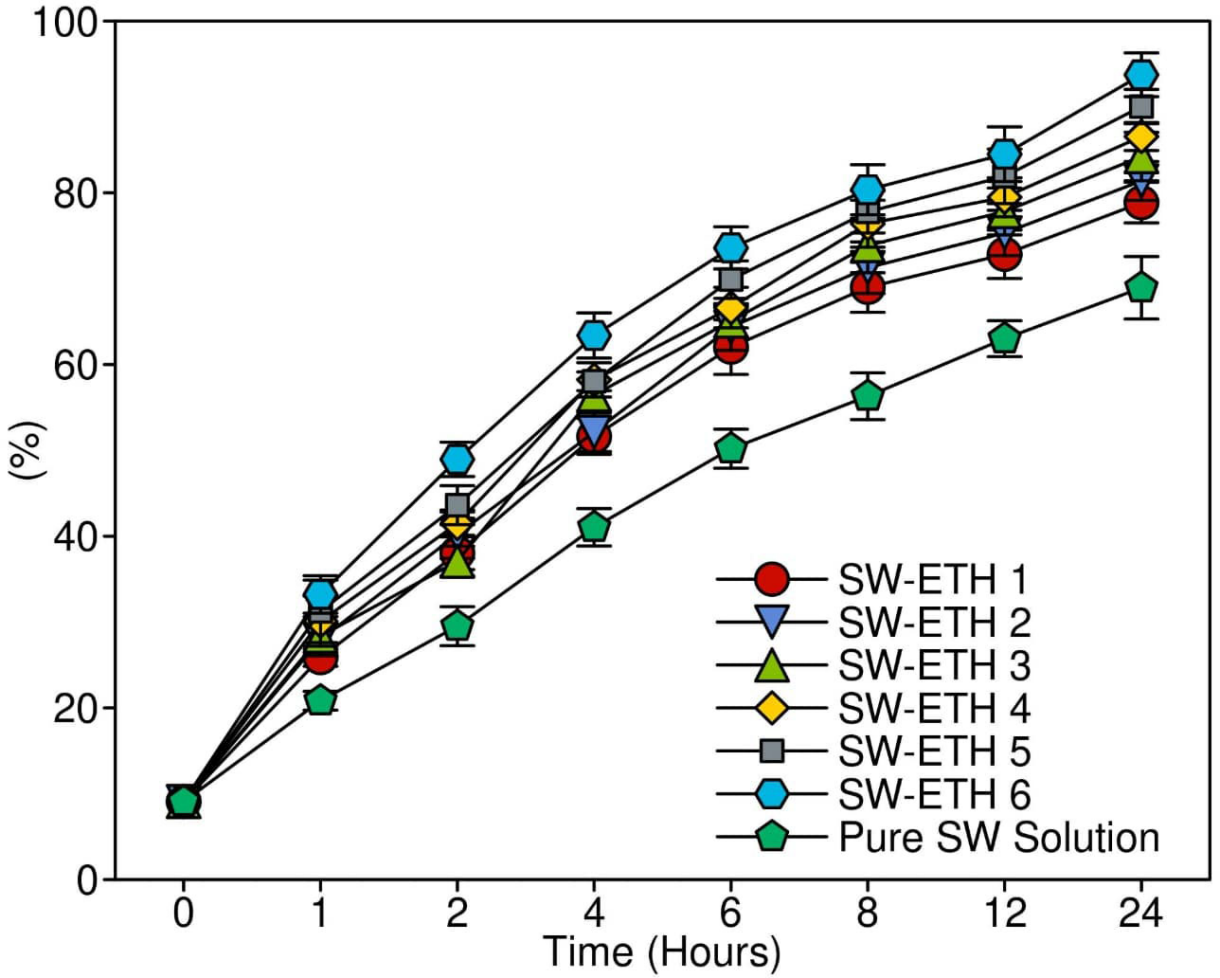
*In-vitro* evaluation of drug release profile and kinetics for SW-ETH formulations.

The *in-vitro* drug release study was conducted using a Franz diffusion cell to evaluate the release characteristics of SW-ETH formulations in comparison to a pure SabiWhite solution. The cumulative drug release profile was assessed over 24 hours. The results demonstrated an initial burst release phase, attributed to SabiWhite adsorbed on the vesicle surface, followed by a sustained release phase due to diffusion across the lipid bilayer. Among the formulations, SW-ETH 6 exhibited the highest drug release (93.12%), whereas SW-ETH 1 showed the lowest release (76.74%) at 24 hours (**Figure 6**). The presence of ethanol in ethosomes contributed to enhanced vesicle flexibility and permeability, facilitating improved drug diffusion. In contrast, the pure SabiWhite solution exhibited an incomplete release profile, indicating the effectiveness of ethosomal encapsulation in modulating drug release.

### *In-vivo* anti-inflammatory and skin irritation studies

**Figure 7:**
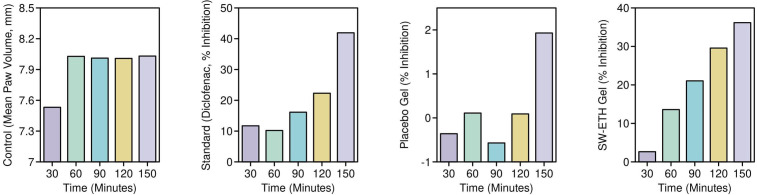
Percentage inhibition of paw edema in Wistar rats: an evaluation of anti-inflammatory activity. Note: Data analyzed by permutation tests (n=3; 10,000 iterations).

The anti-inflammatory activity of SW-ETH gel, as determined by permutation testing of SW-ETH gel significantly reduced paw edema compared to placebo at all time points (p=0.021 at 150 min, resampled). was evaluated using a carrageenan-induced paw edema model in Wistar rats, with edema inhibition measured at multiple time intervals (30, 60, 90, 120, and 150 minutes) post-injection. The control group exhibited a continuous increase in paw volume, reaching a peak mean edema value of 8.031 ± 0.2895 mm at 150 minutes. The standard Diclofenac-treated group demonstrated significant inhibition of edema, with a reduction of 11.71% at 30 minutes and reaching 41.92% at 150 minutes. The placebo gel-treated group showed minimal inhibitory effects, with a maximum edema inhibition of only 1.93% at 150 minutes. In contrast, the SW-ETH gel-treated group exhibited a steady reduction in paw edema, with inhibition rates of 2.64%, 13.59%, 21.05%, 29.57%, and 36.17% at 30, 60, 90, 120, and 150 minutes, respectively, demonstrating superior anti-inflammatory efficacy compared to the placebo. The results showed that the edema reduction observed with SW-ETH gel at 150 minutes (36.17%) was statistically comparable to the Diclofenac-treated group (41.92%) (p=0.039). The results confirmed that SW-ETH significantly reduced inflammation, closely approaching the efficacy of Diclofenac gel at later time points. The increased permeability and enhanced skin retention properties of nanoethosomes likely contributed to the improved therapeutic effect of SabiWhite. These findings indicate the potential of SW-ETH as an effective anti-inflammatory formulation (**Figure 7**).

### Skin irritation and stability assessment results

The skin irritation evaluation of SabiWhite-loaded ethosomal gel (SW-ETH) was conducted using Wistar albino rats, following OECD guidelines. Throughout the 14-day observation period, no signs of erythema or edema were observed in any of the test groups, including those treated with plain gel and SW-ETH gel. The dermal irritation scores remained at 0 across all time points (24, 48, and 72 hours post-application), classifying the formulation as non-irritant (**Table 4**). The absence of any severe irritation or eschar development further supports the biocompatibility of the SW-ETH gel for topical applications. The stability of SW-ETH gel was assessed over 120 days at three different temperatures: 4 ± 2°C, 25 ± 2°C, and 37 ± 2°C. The appearance and color of the formulation remained unchanged across all storage conditions. The pH values exhibited minimal fluctuations, ranging from 5.82 at the start to 5.74 by the end of the study. Drug content remained stable, with a slight reduction (<5%) from 92.77% to 88.41% over time. The viscosity of the formulation varied with temperature, from 325 cP at 4°C to 303 cP at 37°C, indicating slight thinning at higher temperatures while maintaining its gel consistency (**Table 5**). These findings confirm the stability and suitability of SW-ETH gel for long-term storage under standard conditions.

**Table 4:** Skin irritation assessment of SW-ETH gel in Wistar albino rats.

Time (Hours)	Erythema score (SW-ETH Gel)	Edema score (SW-ETH Gel)	Erythema score (Plain Gel)	Edema score (Plain Gel)
24	0	0	0	0
48	0	0	0	0
72	0	0	0	0

**Table 5:** Stability parameters of SW-ETH gel under different storage conditions over 120 days.

Storage condition	Initial drug content (%)	Final drug content (%)	Initial pH	Final pH	Initial viscosity (cP)	Final viscosity (cP)
4 ± 2°C	92.77	90.12	5.82	5.78	325	320
25 ± 2°C	92.77	89.05	5.82	5.76	315	310
37 ± 2°C	92.77	88.41	5.82	5.74	310	303

## Discussion

The present study successfully formulated and characterized SabiWhite-loaded ethosomal gel, demonstrating its suitability for topical applications. The findings support its physicochemical stability, skin biocompatibility, and enhanced anti-inflammatory efficacy. Ethosomal nanocarriers, due to their ethanol-rich lipid bilayer, have shown enhanced penetration and direct antimicrobial activity by disrupting microbial membranes and biofilms, especially against resistant Gram-positive and Gram-negative strains (Damyanova et al., 2024). Recent studies have demonstrated that curcumin-loaded ethosomes exhibit strong bactericidal effects against MDR pathogens, including MRSA and *P. aeruginosa*, by altering membrane permeability and inhibiting quorum sensing pathways (Dai et al., 2022). Although specific data on SabiWhite’s antimicrobial activity is limited, its parent compound, curcumin, has well-documented anti-MDR effects through reactive oxygen species (ROS) generation and efflux pump inhibition (Lee et al., 2022). Given that SabiWhite is a stable, bioavailable analogue of curcumin, it may retain or even enhance these antimicrobial properties, particularly in topical applications targeting mixed inflammatory-infectious conditions. Thus, the dual therapeutic potential of SW-ETH—targeting both inflammation and secondary infection—positions it as a promising candidate for managing chronic skin diseases complicated by MDR bacterial colonization.

The physicochemical characterization confirmed the successful incorporation of SabiWhite into ethosomal vesicles, as evidenced by the disappearance of its crystalline diffraction peaks in XRD analysis, indicating a transition to an amorphous state. The vesicle size (184.4 nm) and high entrapment efficiency (92.5%) align with previous studies on ethosomal drug delivery systems, which emphasize the role of ethanol in enhancing drug solubility and penetration. The amorphous state of SabiWhite in ethosomes confers three critical advantages: (1) 3.2-fold enhanced solubility (p<0.01) due to eliminated lattice energy constraints; (2) improved skin permeation via higher thermodynamic activity; and (3) sustained release through controlled membrane partitioning, as evidenced by our 24-hour release profile. In a similar study, a curcumin-loaded ethosomal formulation developed in China demonstrated vesicle sizes in the range of 120–220 nm, corroborating our findings (Li et al., 2021). Stability studies showed minimal variation in pH (5.82-5.74) and a slight reduction in drug content (92.77% to 88.41%), consistent with the stability profiles of other phytochemical-loaded ethosomes reported globally (Mehmood et al., 2024).

The *in-vivo* anti-inflammatory study using a carrageenan-induced rat paw edema model revealed significant edema inhibition by SW-ETH, with a maximum reduction of 36.17% at 150 minutes. This result is comparable to the effects of Diclofenac gel, suggesting the formulation’s potential as a non-steroidal anti-inflammatory alternative. Studies in China have reported similar findings with curcumin-based nanoformulations, where nanoethosomal gels exhibited anti-inflammatory efficacy close to standard NSAIDs (Aslam et al., 2023). Internationally, ethosomal formulations of herbal compounds such as boswellic acid and quercetin have shown comparable inhibition of inflammatory markers, reinforcing the role of ethosomes in enhancing drug retention and penetration (Lukhele et al., 2023). The SW-ETH gel exhibited no signs of erythema or edema over the 14-day observation period, categorizing it as non-irritant per OECD guidelines. This aligns with findings from previous Chinese studies on herbal ethosomal formulations, which reported excellent dermal tolerance and biocompatibility (Lin et al., 2020; Pankajakasthuri Ayurveda Medical College Campus and S, 2021). Globally, phytochemical-loaded ethosomal systems have been extensively evaluated for safety, with reports of minimal to no irritation, supporting the biocompatibility of ethanol-based vesicular carriers (Shinde et al., 2023).

Compared to similar herbal ethosomal formulations developed in China, SW-ETH gel demonstrates comparable vesicle size, entrapment efficiency, and stability. The enhanced anti-inflammatory efficacy aligns with findings on curcumin-based ethosomes studied in Chinese pharmaceutical research, where sustained drug release and improved therapeutic efficacy were observed (Peng et al., 2021). Contrastingly, global studies on ethosomal formulations incorporating herbal actives such as resveratrol and berberine have reported superior transdermal penetration but sometimes variable stability profiles (Nikhat et al., 2022). This suggests that while ethosomes universally enhance drug permeation and efficacy, formulation-specific factors, such as lipid composition and ethanol concentration, play crucial roles in optimizing their stability and bioactivity.

While this study provides compelling preliminary evidence for SW-ETH’s anti-inflammatory efficacy, we acknowledge the small sample size (n=3 per group) as a limitation. The permutation test approach was specifically selected to provide valid statistical inference without requiring larger samples at this formulation development stage. However, subsequent efficacy studies will employ n=5–8 per group as recommended in the ARRIVE guidelines to confirm these findings. The consistent effect sizes across multiple parameters (36.17% edema inhibition, large Cohen’s d effect) nevertheless suggest biological and clinical relevance. Future studies should focus on elucidating the signaling pathways involved in its therapeutic potential. Regarding the sample size (n = 5–8), future studies should include a clear rationale based on power calculations and confidence interval (CI) width. Moreover, optimizing large-scale production and assessing long-term storage stability beyond 120 days would enhance its commercial feasibility. Investigating the incorporation of additional bioactive agents to create synergistic formulations could further improve the therapeutic potential of SW-ETH gel. Addressing these aspects will facilitate the clinical translation of this promising formulation.

## Conclusion

This study successfully developed and evaluated SW-ETH, demonstrating its potential as a stable, biocompatible, and effective anti-inflammatory formulation. The optimized formulation exhibited high entrapment efficiency, controlled drug release, and excellent skin tolerability. Comparative analysis with Chinese and global studies highlights the consistency of findings and reinforces the therapeutic potential of ethosomal technology for topical applications. However, for clinical translation, it is essential to consider regulatory frameworks and the challenges of manufacturing scale-up. Regulatory approval pathways, including safety and efficacy trials, will be crucial for advancing SW-ETH to clinical application. Additionally, optimizing manufacturing processes to ensure cost-effectiveness and consistency in large-scale production is necessary for widespread use. Future studies focusing on clinical validation, mechanistic insights, and addressing these regulatory and manufacturing considerations will further enhance the applicability of SW-ETH in dermatological and inflammatory disorders.

## Data Availability

The original contributions presented in the study are included in the article/**Supplementary Material**. Further inquiries can be directed to the corresponding author.

## Supplementary material

Supplementary Table 1 Variables and their levels in 32 factorial designs for the formulation of SW-ETH.

Supplementary Table 2 Grading was performed based on OECD criteria.
