# Supplementary material for: Development and optimization of a novel nanocarrier SabiWhite-loaded ethosomal gel for targeted skin inflammation complicated by multidrug-resistant pathogens
Source: Front Cell Infect Microbiol. 2025 Jul 22;15:1640799. doi: 10.3389/fcimb.2025.1640799 (PMC12321823; doi:10.3389/fcimb.2025.1640799)
Supplement: Supplementary Table 1 — Variables and their levels in 32 factorial designs for the formulation of SW-ETH. [file Table1.docx]

**S-1:** **Variables and their levels in 32 factorial designs for the formulation of Sw-ETH**

| **Factor** | **Levels used, actual (coded)** | | |
| --- | --- | --- | --- |
|  | **LOW (-1)** | **MEDIUM (0)** | **HIGH (+1)** |
| **Independent variables** |  | | |
| X_1_: Phospholipon -90G (% w/v) | 2 | 3 | 4 |
| X_2_: Ethanol (% v/v) | 25 | 35 | 45 |
| **Dependent variables** |  | | |
| Y_1_: Vesicle size (nm) | In the range (100-200) | | |
| Y_2_: Entrapment efficiency (%) | Maximize | | |

**S-2** **Grading was performed based on OECD criteria**

| **Erythema and Eschar Formation** | **Score** |
| --- | --- |
| No erythema | 0 |
| Very slight erythema | 1 |
| Well-defined erythema | 2 |
| Moderate to severe erythema | 3 |
| Severe erythema/eschar formation | 4 |
